# Role of an Exosomes-Related lncRNAs Signature in Tumor Immune Microenvironment of Gastric Cancer

**DOI:** 10.3389/fcell.2022.873319

**Published:** 2022-04-06

**Authors:** Chan Li, Zeyu Zhang, Emin Peng, Jinwu Peng

**Affiliations:** ^1^ Department of Cardiovascular Medicine, Xiangya Hospital, Central South University, Changsha, China; ^2^ Department of Thyroid Surgery, Xiangya Hospital, Central South University, Changsha, China; ^3^ Xiangya International Medical Center, Xiangya Hospital, Central South University, Changsha, China; ^4^ National Clinical Research Center for Geriatric Disorders, Xiangya Hospital, Central South University, Changsha, China; ^5^ Department of Pathology, Xiangya Hospital, Central South University, Changsha, China; ^6^ Department of Pathology, Xiangya Changde Hospital, Changde, China

**Keywords:** gastrointestinal cancer, exosome, lncRNA, ncRNA, tumor microenvironment

## Abstract

**Background:** Exosomes plays a crucial role in intercellular communication of gastric cancer (GC), while long non-coding RNAs (lncRNAs) contributes to the tumorigenesis and progression of GC. This study aims to explore the prognostic exosomes-related lncRNAs of GC patients.

**Methods:** Data of 375 GC patients were obtained from the TCGA database. The entire cohort was randomly divided into a training cohort and a validation cohort in a 2:1 ratio. Exosomes-related lncRNAs were identified by the Pearson correlation analysis with reported exosomes-related genes. LASSO Cox regression was used to construct the signature.

**Results:** A prognostic signature consisting of 11 exosomes-related lncRNAs was identified, and patients with lower risk scores had a better prognosis than those with higher risk scores. ROC curves and multivariate Cox regression analysis showed that the signature was an independent risk factor for prognosis in both the training (HR: 3.254, 95% CI: 2.310–4.583) and validation cohorts (HR: 1.974, 95% CI: 1.108–3.517). Gene set enrichment analysis (GSEA) suggested associations between the signature and several immune-related pathways. The identified signature was shown to be associated with GC tumor microenvironment. The expression of two immune checkpoints was also increased in the high-risk group, including B7-H3 and VSIR, indicating the potential role of the identified signature in GC immunotherapies.

**Conclusion:** A novel exosomes-related lncRNA signature, which may be associated with tumor immune microenvironment and potentially serve as an indicator for immunotherapy, has been identified to precisely predict the prognosis of GC patients.

## Background

Gastric cancer (GC) is one of the most prevalent malignant tumor types in digestive system, and the fourth leading cause of cancer-related death worldwide, accounting for 7.7% of all cancer deaths (Liu et al., 2021; Sung et al., 2021). Although mortality has decreased in recent years due to the developments of earlier screening and immunotherapies, the prognosis of GC patients remains low, with a 5-year survival rate of 32% (Niclauss et al., 2021). Moreover, due to the individual heterogeneity, the criteria of treatment selection and high-risk patients recognization are still lacking. Thus, exploring reliable prognostic biomarkers is vital to preferable individualized management and treatment.

Exosomes constitute a set of tiny extracellular vesicles with an approximate diameter of 30–100 nm, which have been proven to play a crucial role in intercellular communication through the intercellular transfer of nucleic acids and specific repertoires of proteins and lipids (Kaushik and Cuervo, 2015; Phan et al., 2018; Kumar et al., 2019). Multiple studies have reported the role of exosomes in both physiological and pathological processes, including various types of cancer (Thakur et al., 2020; Thakur et al., 2022). Particularly, the biological roles of exosomes in GC have been preliminarily clarified by many researches, involving in GC progression, immune escape and metastasis (Fu et al., 2019).

With advances in sequencing technology, long non-coding RNAs (lncRNAs) have been found to be functional in most biological and pathological processes. Evidence has indicated potential contributions of lncRNAs in the tumorigenesis and progression of GC (Yuan et al., 2020). However, the role of exosomes-related lncRNAs in GC pathogenesis and immune regulation remains underappreciated. Thus, this study was performed to recognize the prognostic exosomes-related lncRNAs in GC, thus providing a better understanding of the prognosis prediction and selection of patients for immunotherapy.

## Materials and Methods

### Data Acquisition

Transcriptome and clinical data of GC patients, including 375 tumor tissues and 32 normal tissues, were obtained from the STAD project of TCGA database (http://cancergenome.nih.gov/). Patients without sufficient clinical data, including patient characteristics and survival data, were excluded from this study. Expression data were normalized to transcripts per kilobase million (TPM) for further analysis.

### Identification of Exosomes-Related lncRNAs

120 exosomes-related genes were obtained from a comprehensive database of exosomes (Supplementary Material 1) (Wang et al., 2021). Univariate Cox regression was performed to screen the prognostic exosomes-related genes. Correlations of prognostic exosomes-related genes and lncRNAs were examined by Pearson correlation. Based on the cut-off criteria of Pearson correlation coefficient >0.3, these lncRNAs were considered as candidate exosomes-related lncRNAs.

### Construction and Validation of the Prognostic Exosomes-Related lncRNAs Signature

The entire cohort was randomly divided into two cohorts (training: validation = 2:1), while the randomization was achieved by random numbers produced by Excel. The former was adopted for the construction of the prognostic exosomes-related lncRNAs signature, while the latter for validation. Prognostic exosomes-related lncRNAs was selected by univariate Cox regression analysis, and the signature was constructed by the least absolute shrinkage and selection operator (LASSO) Cox regression in R platform. The prognostic value of the signature was subsequently confirmed by survival analysis, time-dependent ROC curves, and multivariate Cox regression.

### The mRNA-lncRNA Co-Expression Network

We subsequently created a co-expression network and a Sankey diagram, presenting the connection between exosomes-related genes and lncRNAs.

### Gene Set Enrichment Analysis

According to the risk score, we divided tumor tissues into two groups: the low-risk group and high-risk group. Differentially expressed genes (DEGs) were recognized using the “limma” package with cut-off criteria of adjust *p*-value <0.05 and |log_2_foldchange| > 1. Subsequently, these DEGs were uploaded for GSEA analysis (http://www.broadinstitute.org/gsea).

### Immunological Analysis

Tumor IMmune Estimation Resource (TIMER) database was employed to investigate the abundance of tumor-infiltrating immune cells in GC tissues (https://cistrome.shinyapps.io/timer/).

### Statistical Analysis

R 3.3.0 and Statistical Package for Social Sciences 23.0 (SPSS Inc., Chicago, IL, USA) were used for statistical analysis. ANOVA was used to analyze differences in immune cell components between the normal and GC tissues.

## Results

### Training Cohort and Validation Cohort

**FIGURE 1:**
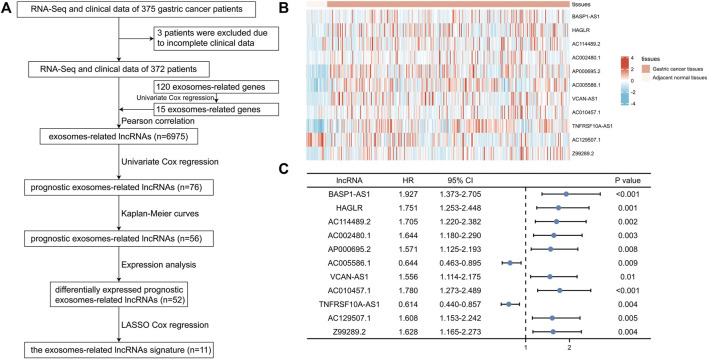
Identification of prognostic exosomes-associated lncRNAs in gastric cancer (GC) patients. **(A)** The flow chart of constructing the exosomes-related lncRNAs signature. **(B)** The heatmap of 11 prognostic exosomes-related lncRNAs in GC tissues and adjacent normal tissues. **(C)** Univariate Cox regression of 11 prognostic exosomes-related lncRNAs. *p < 0.05; **p < 0.01; ***p < 0.001.

Three hundred and seventy-two patients were finally included in the analyses (Figure 1A), with 248 in the training cohort and 124 in the validation cohort. The patient characteristics in the two cohorts were shown in Table 1, with no statistically significant differences.

**TABLE 1:** The characteristics of gastric cancer patients in training cohort and validation cohort.

Characteristic	Training cohort (*n* = 248)	Validation cohort (*n* = 124)	*p* value
Age, median (IQR)	67 (58, 72)	69 (58, 75)	0.144
Gender, *n* (%)			0.516
Female	92 (37.1%)	41 (33.1%)	
Male	156 (62.9%)	83 (66.9%)	
Race, *n* (%)			0.982
Asian	49 (22.7%)	24 (22.4%)	
Black or African American	8 (3.7%)	3 (2.8%)	
White	158 (73.1%)	80 (74.8%)	
Other	1 (0.5%)	0 (0%)	
Neoplasm histologic grade, *n* (%)			0.928
G1	7 (2.9%)	3 (2.5%)	
G2	91 (37.6%)	43 (35.5%)	
G3	144 (59.5%)	75 (62%)	
Clinical stage, *n* (%)			0.186
Stage I	39 (16.5%)	11 (9.8%)	
Stage II	71 (30%)	40 (35.7%)	
Stage III	98 (41.4%)	52 (46.4%)	
Stage IV	29 (12.2%)	9 (8%)	
T stage, n (%)			0.390
T1	13 (5.3%)	5 (4.2%)	
T2	58 (23.8%)	20 (16.7%)	
T3	107 (43.9%)	61 (50.8%)	
T4	66 (27%)	34 (28.3%)	
N stage, *n* (%)			0.328
N0	78 (32.5%)	30 (26.3%)	
N1	69 (28.7%)	28 (24.6%)	
N2	47 (19.6%)	28 (24.6%)	
N3	46 (19.2%)	28 (24.6%)	
M stage, *n* (%)			0.775
M0	220 (92.4%)	108 (93.9%)	
M1	18 (7.6%)	7 (6.1%)	

### Identification of Prognostic Exosomes-Related lncRNAs

Among the 120 exosomes-related genes, 15 were shown to have prognostic value in GC (Figure 1A). 6,975 lncRNAs were identified to be related with these prognostic exosomes-related genes. Univariate Cox regression, Kaplan-Meier curves, and expression analyses were conducted to varify the prognostic value of these lncRNAs and 52 were recognized as prognostic exosomes-related lncRNAs. 11 prognostic exosomes-related lncRNAs (BASP1-AS1, HAGLR, AC114489.2, AC002480.1, AP000695.2, AC005586.1, VCAN-AS1, AC010457.1, TNFRSF10A-AS1, AC129507.1, Z99289.2) were screened to participate in the prognostic signature by LASSO Cox regression analysis. The prognostic signature was presented as follows: risk score = (0.3008 * BASP1-AS1 expression) + (0.0184 * HAGLR expression) + (0.3687 * AC114489.2 expression) + (0.3893 * AC002480.1 expression) + (0.0687 * AP000695.2 expression) + (−0.0375 * AC005586.1 expression) + (0.0025 * VCAN-AS1 expression) + (0.1462 * AC010457.1 expression) + (−0.0008 * TNFRSF10A-AS1 expression) + (0.1595 * AC129507.1 expression) + (0.2522 * Z99289.2 expression). The transcriptional expression of the 11 lncRNAs in GC were shown by heatmaps in Figure 1B. Their prognostic values were shown in Figure 1C with the univariate Cox regression.

**FIGURE 2:**
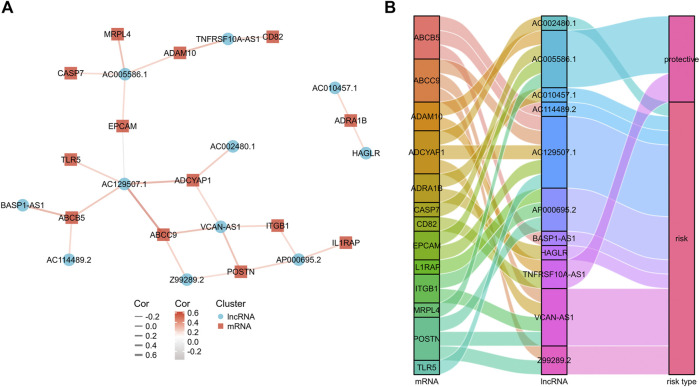
The mRNA-lncRNA co-expression network. **(A)** mRNA-lncRNA co-expression network of the exosomes-related genes and the selected exosomes-related lncRNAs. **(B)** The Sankey diagram showing the connection degree between the exosomes-related lncRNAs and the exosomes-related genes.

Then, a co-expression network between the prognostic exosomes-related genes and lncRNAs was constructed to confirm their relationships. As shown in Figure 2A, 13 prognostic exosomes-related genes and 11 lncRNAs were included in the network. Particularly, AC129507.1, VCAN-AS1, and AC005586.1 were widely associated with multiple exosomes-related genes. In addition, the close correlation between these genes and lncRNAs was indicated by the Sankey diagram (Figure 2B). These findings suggest that the 11 exosomes-related lncRNAs may play important roles in GC.

### Validation of the Prognostic Exosomes-Related lncRNA Signature

**FIGURE 3:**
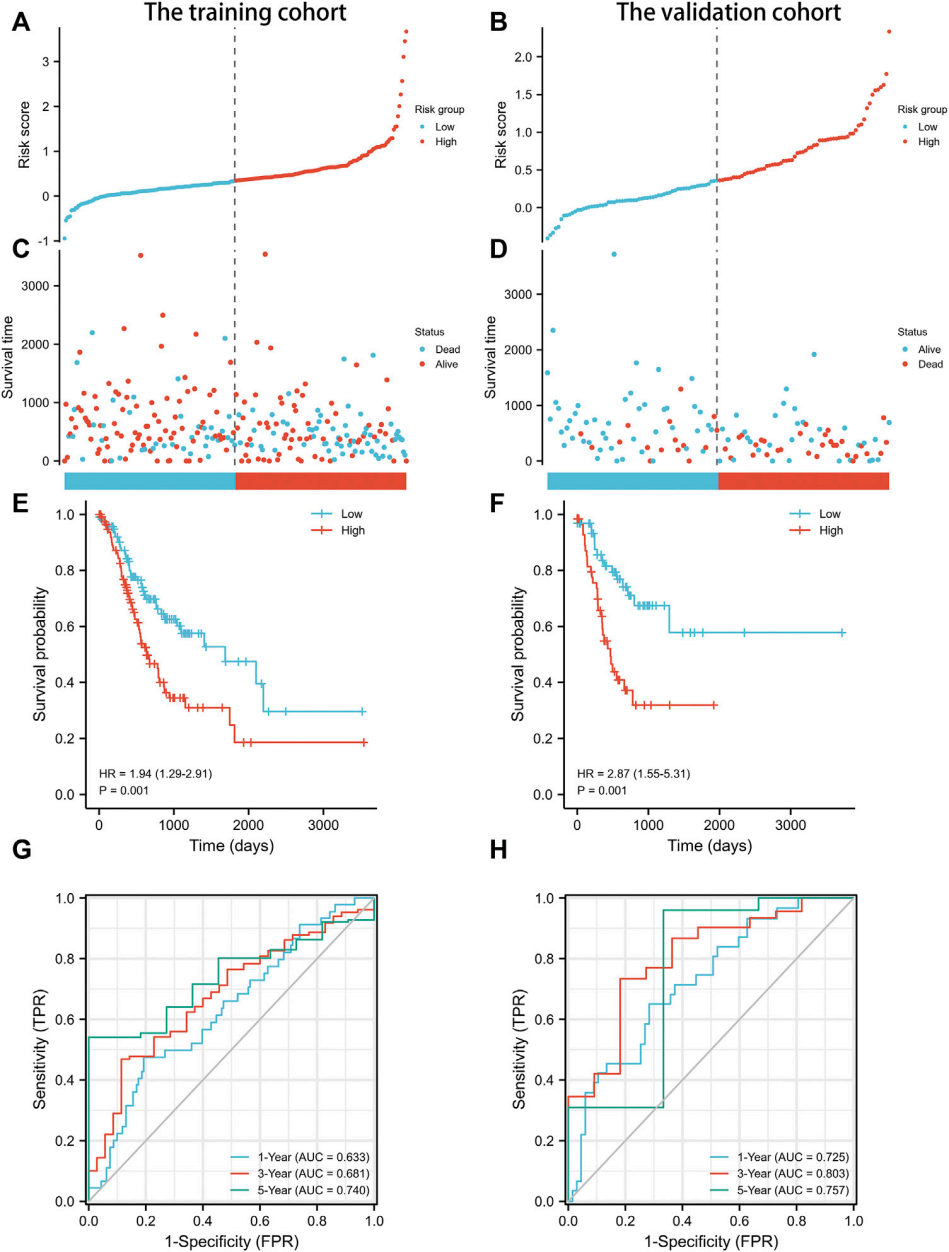
Prognostic analysis of exosomes-related lncRNA signature in the training cohort and validation cohort. **(A)** The distribution of the risk scores in the training cohort. **(B)** The distribution of the risk scores in the validation cohort. **(C)** The distributions of overall survival status, overall survival, and risk score in the training cohort. **(D)** The distributions of overall survival status, overall survival, and risk score in the validation cohort. **(E)** Kaplan-Meier curves for the overall survival of patients in the high- and low-risk groups in the training cohort. **(F)** Kaplan-Meier curves for the overall survival of patients in the high- and low-risk groups in the validation cohort. **(G)** AUC of time-dependent ROC curves verified the prognostic accuracy of the risk score in the training cohort. **(H)** AUC of time-dependent ROC curves verified the prognostic accuracy of the risk score in the validation cohort.

The prognostic signature was subsequently validated in two cohorts. The patients in the two cohorts were divided into the low-risk group and high-risk group based on the median risk score. Figures 3A–D showed that the probability of death was higher in the high-risk group than in the low-risk group in both cohorts. Kaplan-Meier curves of OS showed consistent results in both cohorts (Figures 3E,F), indicating that the survival of GC patients in the high-risk group was significantly worse than that in the low-risk group. A time-dependent receiver operating characteristic (ROC) was performed to investigate the prognosis value of the signature (Figures 3G,H). The area under the curve (AUC) reached 0.633 at 1-year, 0.681 at 3-year, and 0.740 at 5-year in the training cohort, while 0.725 at 1-year, 0.803 at 3-year, and 0.757 at 5-year in the validation cohort. In order to further verify the prognostic value of the signature, multivariate Cox regression analysis was performed in the training cohort (Table 2) and the validation cohort (Table 3). The results showed that the risk score could be an independent factor to predict the patient prognosis in the two cohorts.

**TABLE 2:** Univariate and multivariate analyses of risk factors with and OS in the training cohort.

Variables	HR (95% CI)	*p* value
Univariate analyses		
Age (years)	1.027 (1.007–1.049)	0.009
Gender (female vs. male)	0.849 (0.557–1.295)	0.448
Race		
Asian vs. Black or African American	0.518 (0.180–1.492)	0.223
White vs. Black or African American	0.719 (0.287–1.803)	0.482
Other vs. Black or African American	5.857 (0.659–52.085)	0.113
Neoplasm histologic grade (G3 vs. G1–2)	1.094 (0.726–1.648)	0.668
Clinical stage (III and IV vs. I and II)	1.580 (1.038–2.405)	0.033
T stage (T3–4 vs. T1–2)	1.493 (0.930–2.398)	0.097
N stage (N2–3 vs. N0–1)	1.609 (1.075–2.410)	0.021
M stage (M1 vs. M0)	1.447 (0.727–2.879)	0.293
Risk score	2.718 (1.975–3.742)	<0.001
Multivariate analyses		
Age (years)	1.039 (1.017–1.063)	<0.001
Clinical stage (III and IV vs. I and II)	0.952 (0.509–1.781)	0.878
T stage (T3–4 vs. T1–2)	2.076 (1.131–3.811)	0.018
N stage (N2–3 vs. N0–1)	1.392 (0.811–2.388)	0.230
Risk score	3.254 (2.310–4.583)	<0.001

HR, hazard ratio; CI, confidence interval; OS, overall survival.

**TABLE 3:** Univariate and multivariate analyses of risk factors with and OS in the validation cohort.

Variables	HR (95% CI)	*p* value
Univariate analyses		
Age (years)	1.017 (0.987–1.047)	0.278
Gender (female vs. male)	0.664 (0.349–1.265)	0.213
Race		
Asian vs. Black or African American	0.475 (0.095–2.363)	0.363
White vs. Black or African American	0.736 (0.176–3.083)	0.675
Neoplasm histologic grade (G3 vs. G1–2)	2.015 (1.033–3.930)	0.040
Clinical stage (III and IV vs. I and II)	2.951 (1.473–5.913)	0.002
T stage (T3–4 vs. T1–2)	2.540 (0.999–6.459)	0.050
N stage (N2–3 vs. N0–1)	1.720 (0.942–3.139)	0.077
M stage (M1 vs. M0)	30.157 (8.642–105.230)	<0.001
Risk score	2.281 (1.442–3.608)	<0.001
Multivariate analyses		
Neoplasm histologic grade (G3 vs. G1–2)	1.351 (0.600–3.041)	0.468
Clinical stage (III and IV vs. I and II)	4.254 (1.440–12.562)	0.009
T stage (T3–4 vs. T1–2)	1.087 (0.352–3.355)	0.885
N stage (N2–3 vs. N0–1)	0.589 (0.229–1.513)	0.271
M stage (M1 vs. M0)	15.469 (3.720–64.326)	<0.001
Risk score	1.974 (1.108–3.517)	0.021

HR, hazard ratio; CI, confidence interval; OS, overall survival.

### Nomogram Based on the Signature for Predicting the OS of Gastric Cancer Patients

**FIGURE 4:**
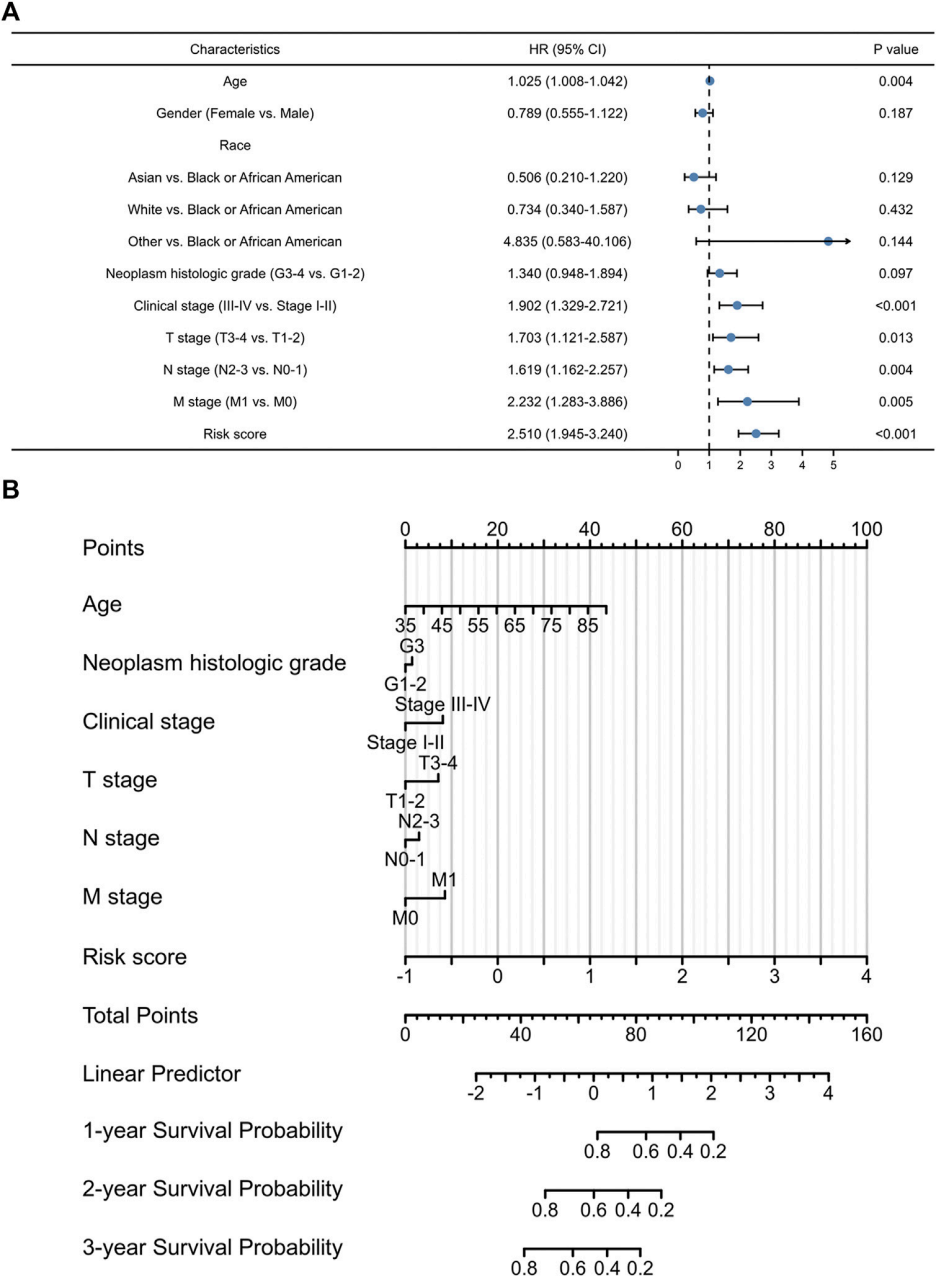
The prognostic values of exosomes-related lncRNA signature. **(A)** Multivariate Cox regression of patient characters and the signature in the whole cohort. **(B)** The nomogram constructed using patient characters and the signature.

In order to develop a more accurate model for prognosis prediction, univariate Cox regression analysis was performed (Figure 4A). A nomogram, consisting of the identified exosomes-related lncRNAs signature and multiple patient characteristics, was proven to be effective for survival probability prediction with a C-index of 0.707 (95% CI: 0.682–0.733) in GC (Figure 4B).

### Associations Between the Signature and Immune-Related Pathways

**FIGURE 5:**
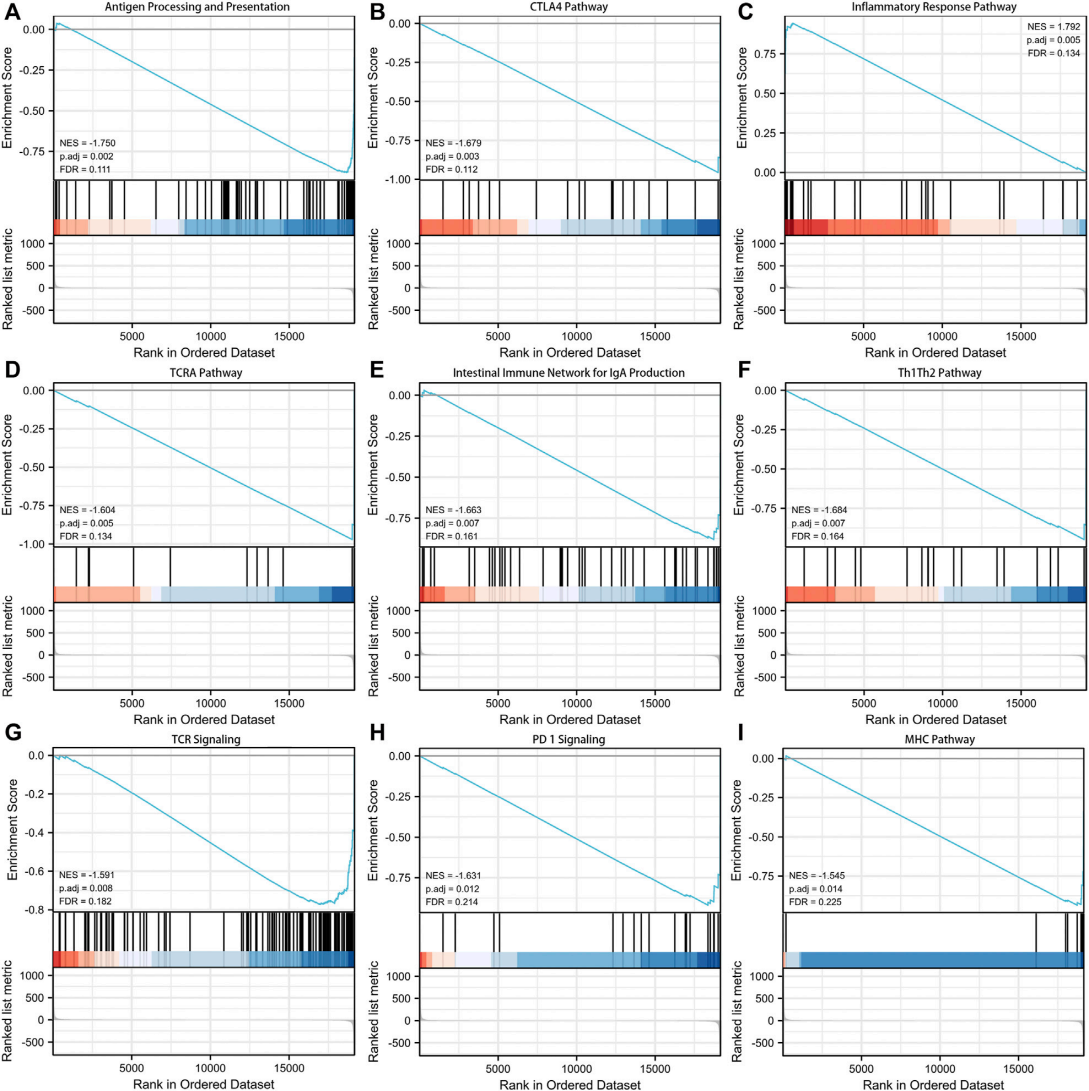
Gene set enrichment analysis (GSEA) about the exosomes-related lncRNA prognostic signature. **(A)** Antigen processing and presentation. **(B)** CTLA4 pathway. **(C)** Inflammatory response pathway. **(D)** TCRA pathway. **(E)** Intestinal immune network for IgA production. **(F)** Th1Th2 pathway. **(G)** TCR signaling. **(H)** PD-1 signaling. **(I)** MHC pathway.

GSEA analysis was performed to explore the potential biological functions involved in the signature. Figure 5 presented the top nine immune-associated signaling pathways, including antigen processing and presentation, CTLA4 pathway, inflammatory response pathway, TCRA pathway, intestinal immune network for IgA production, Th1Th2 pathway, TCR signaling, PD-1 signaling, and MHC pathway. These findings suggested the potential associations between the identified signature and immune regulation.

### Associations Between the Signature and Immune Infiltration

**FIGURE 6:**
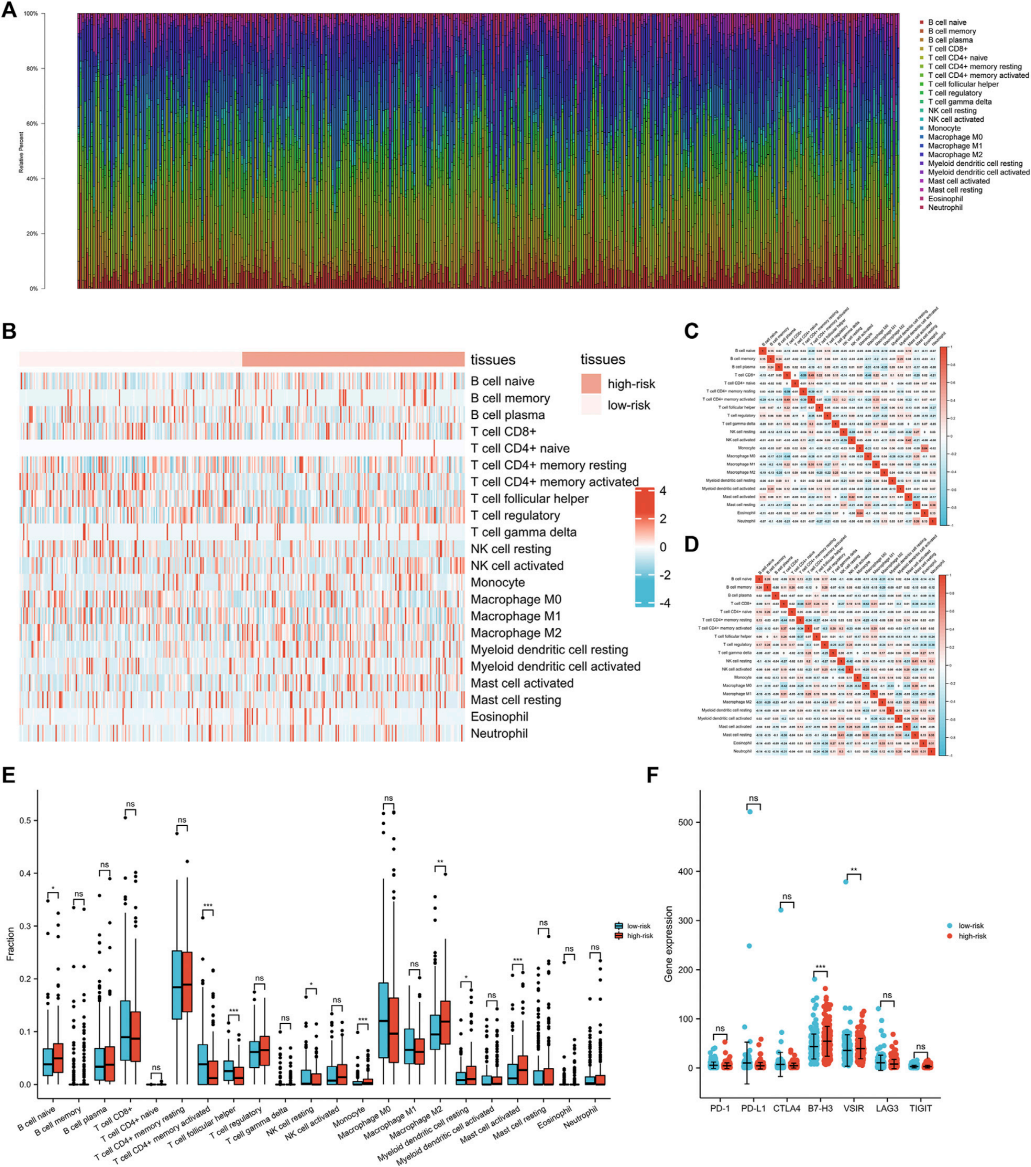
The interactions between exosomes-related lncRNA signature and immune regulation in gastric cancer (GC) patients. **(A)** The barplot of the tumor-infiltrating cell proportions. **(B)** The heatmap of the tumor-infiltrating cell proportions. **(C)** Correlation matrix of immune cell proportions in the low-risk group. **(D)** Correlation matrix of immune cell proportions in the high-risk group. **(E)** Comparisons of immune cell proportions between the low-risk group and the high-risk group. **(F)** Comparisons of multiple immune checkpoints between the low-risk group and the high-risk group, including PD-1, PD-L1, CTLA4, B7-H3, VSIR, LAG3, TIGIT.

To explore the roles of the exosomes-related lncRNA signature in the immune microenvironment of GC patients, the associations between the signature and immune infiltration cells were further investigated using the ESTIMATE algorithm. The proportions of various immune cells were shown in Figures 6A,B, and the correlations between these immune cells in the low-risk and high-risk groups were shown in Figures 6C,D. Among the immune cells, naive B cell, monocyte, M2 macrophages, resting myeloid dendritic cell, and activated mast cell were increased in the high-risk group, while the CD4^+^ memory activated T cells, follicular helper T cell, and resting NK cell were decreased in the high-risk group (Figure 6E). The correlations between the signature and various immune checkpoints were also investigated. As shown in Figure 6F, B7-H3 and VSIR were upregulated in the high-risk group, suggesting their potential value in GC immunotherapy.

## Discussion

In this study, prognostic exosomes-related lncRNAs in GC were comprehensively investigated. A novel prognostic signature consisting of 11 exosomes-related lncRNAs (BASP1-AS1, HAGLR, AC114489.2, AC002480.1, AP000695.2, AC005586.1, VCAN-AS1, AC010457.1, TNFRSF10A-AS1, AC129507.1, Z99289.2) was constructed using LASSO Cox regression analysis and validated by various analyses. The subsequent functional analysis confirmed the associations between the signature and multiple immune-related pathways. The results suggested the potential value of the identified signature in predicting patient prognosis and managing immunotherapies.

Several studies have investigated the roles of exosomes-related proteins in GC (Thakur et al., 2021). TGF-β1 was reported in plasma exosomes of GC patients, and exosomal level of TGF-β1 was correlated with lymphatic metastasis of GC (Yen et al., 2017). Yoon et al. (2018) reported that GKN1, a crucial protein in mucosal homeostasis, was found in exosomes and could be internalized by the gastric epithelium, which might play an important role in gastric tumorigenesis protection. Particularly, the genes reported in this study were also partially recognized in GC. ADAM10 was reported to be associated with lymph node and distant metastasis, and poor prognosis in GC (Wang et al., 2011). A meta-analysis with 11 included studies by Dai et al. (2017) demonstrated that EPCAM was associated with larger tumour size, lymph node metastasis and worse prognosis in GC. Furthermore, Hu et al. (2017) found that ITGB1 could be a downstream factor of hTERT, thus promoting the invasion of GC cells. However, detailed investigations about the roles of these proteins in exosomes of GC are still lacking.

The role of lncRNAs was preliminarily investigated in exosomes of GC. Exosomal HOTTIP was reported to be associated with poor overall survival (Zhao et al., 2018), while exosomal ZFAS1 was associated with lymph node metastasis and poorer clinical stage (Pan et al., 2017). Particularly, among the 11 identified exosomes-related lncRNAs, VCAN-AS1 was previously found to contribute to the progression of GC *via* regulating p53 expression (Feng et al., 2020). More recently, Hu et al. (2022) reported that lncRNA HAGLR could sponge miR-338-3p to promote 5-Fu resistance through targeting the LDHA-glycolysis pathway in GC. However, the role of these two lncRNAs in exosomes of GC have not been demonstrated. Meanwhile, the other identified 9 lncRNAs are also poorly investigated in GC.

Our study shows significant relationships between the signature and many immune-associated signaling pathways, strongly suggesting its potential role in GC immunotherapy. Pembrolizumab and nivolumab (targeting PD-1) and ipilimumab (targeting CTLA-4) have been applied in GC treatment with encouraging anti-tumor results (Joshi and Badgwell, 2021). In this study, two immune checkpoints (B7-H3 and VSIR), which were previously reported to be attractive targets for immnuotherapy in different cancers (Huang et al., 2020; Bolandi et al., 2021), were upregulated in high-risk patients. These results indicated that B7-H3 and VSIR may be novel checkpoints for developing immunotherapies in GC. In addition, The identified signature in this study provided foundations for selecting B7-H3 and VSIR therapy-sensitive GC patients.

This study surely had some limitations. Firstly, other cohorts other than those from TCGA database were needed for further validations in GC. Secondly, the underlying immune-related mechanisms of the identified lncRNAs were not investigated in this study. Moreover, *in vitro* and *in vivo* experiments were demanded to validate our results.

## Conclusion

In summary, a novel exosomes-related lncRNA signature has been identified to precisely predict the prognosis of GC patients. The signature is robustly connected to the regulation of tumor immune microenvironment, providing a personalized prediction model for the prognosis and immunotherapeutic response of GC patients.

## Data Availability

The datasets presented in this study can be found in online repositories. The names of the repository/repositories and accession number(s) can be found in the article/Supplementary Material.

## Supplementary Material

https://www.frontiersin.org/articles/10.3389/fcell.2022.873319/full#supplementary-material

## Abbreviations

ANOVA, One-way analysis of variance; AUC, area under curve; GC, gastric cancer; GSEA, gene set enrichment analysis; lncRNAs, long non-coding RNAs; LASSO, least absolute shrinkage and selection operator; TIMER, Tumor IMmune Estimation Resource; TPM, transcripts per kilobase million.
